# Supplementary material for: Salt-Induced Membrane-Bound Conformation of the NAC Domain of α-Synuclein Leads to Structural Polymorphism of Amyloid Fibrils
Source: Biomolecules. 2025 Mar 31;15(4):506. doi: 10.3390/biom15040506 (PMC12024755; doi:10.3390/biom15040506)
Supplement: Supplementary file 1 [file biomolecules-15-00506-s001.zip › biomolecules-3535169-supplementary.pdf]

biomolecules

Supporting Information

Salt-Induced Membrane-Bound Conformation of the NAC Domain of α-synuclein Leads to Structural Polymorphism of Amyloid Fibrils

Ryota Imaura [1] and Koichi Matsuo [1,2,3,4,*]

[1] Graduate School of Advanced Science and Engineering, Hiroshima University, Hiroshima 739-8511, Japan
[2] Research Institute for Synchrotron Radiation Science, Hiroshima University, Hiroshima 739-0046, Japan
[3] International Institute for Sustainability with Knotted Chiral Meta Matter (WPI-SKCM[2]), Hiroshima University, Hiroshima 739-8526, Japan
[4] Research Institute for Semiconductor Engineering, Hiroshima University, Higashi-Hiroshima 739-8527, Japan

Figures S1 to S6

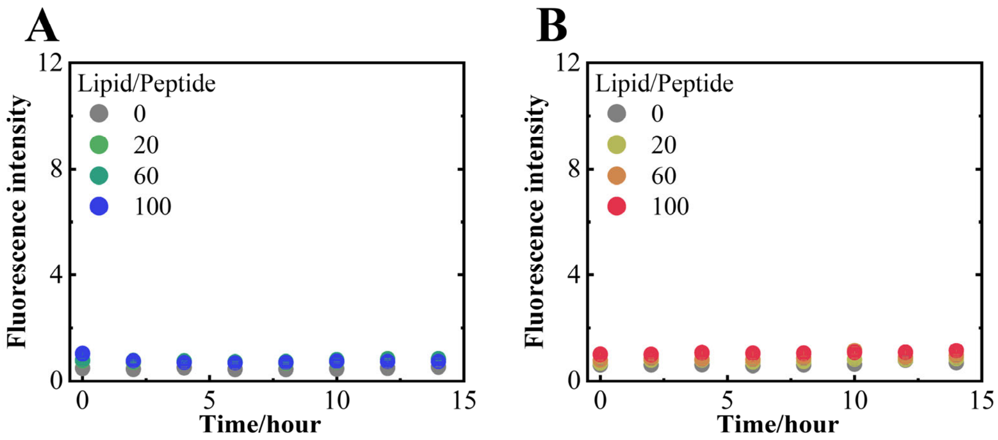

Figure S1. ThT fluorescence at L/P ratios of 0, 20, 60, 100 in the absence (**A**) or presence (**B**) of NaCl. The concentrations of ThT and αS were 10 μM and 50 μM, and the lipid membrane was DMPC.

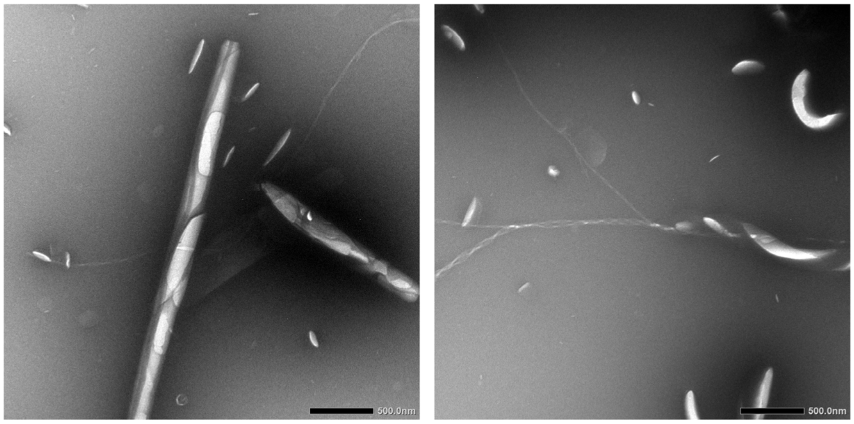

Figure S2. TEM images of amyloid fibrils at the L/P=100 in presence of NaCl. The scale bars represent 500 nm.

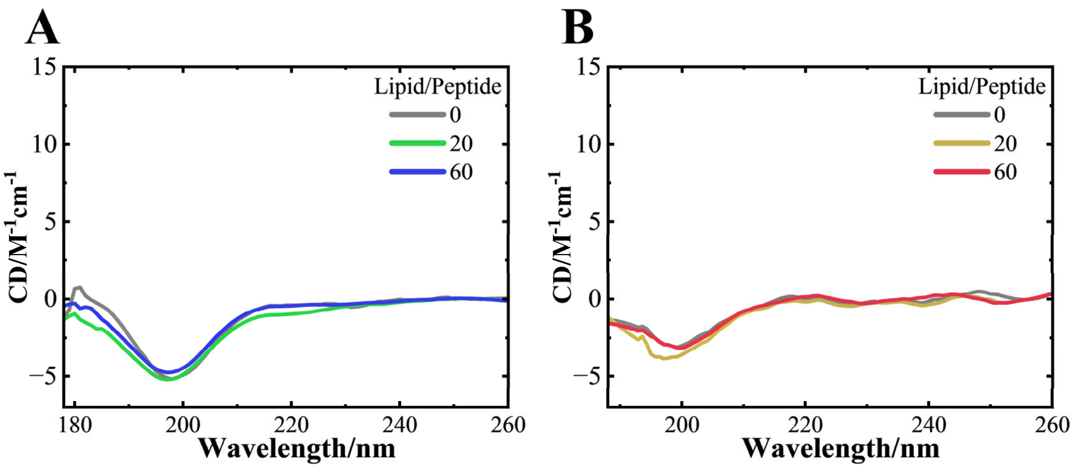

Figure S3. SRCD spectra of $\alpha S_{57\text{-}102}$ at various L/P ratios in the (**A**) absence and (**B**) presence of 0.1 M NaCl. The concentration of $\alpha S_{57\text{-}102}$ was 50 μM, and the lipid membranes were composed of DMPC.

	α-Helix Contents (%)	Number
AlphaFold2	73.9	1
JPred	67.4	1
PSIPRED	76.1	3
SOPMA	41.3	4

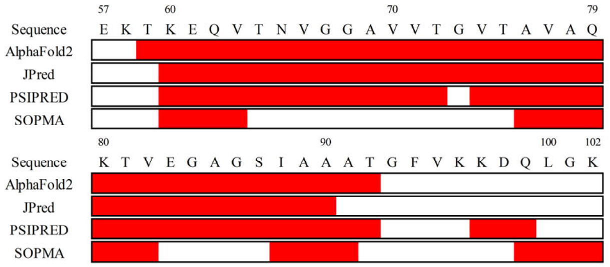

Figure S4. Predicted α-helix content (**Left**) and secondary structure positions (**Right**) from several sequence-based secondary-structure predictions. In the right figure, from top to bottom: residue number, amino-acid residue, and secondary structure positions of $\alpha S_{57\text{-}102}$. Red represents the positions of α-helix segments.

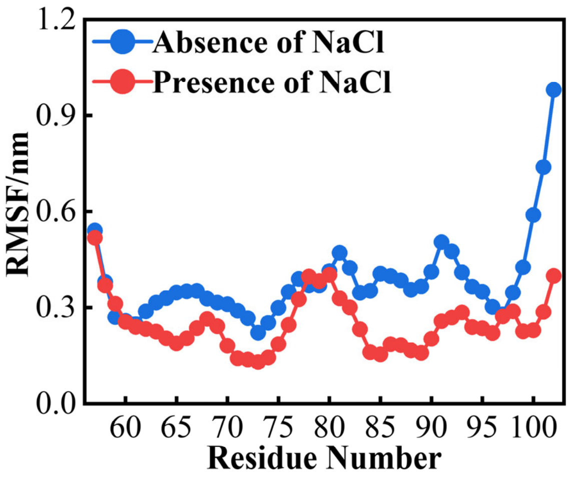

Figure S5. The RMSF results of amino acid residues in $\alpha S_{57\text{-}102}$ (490–500 ns).

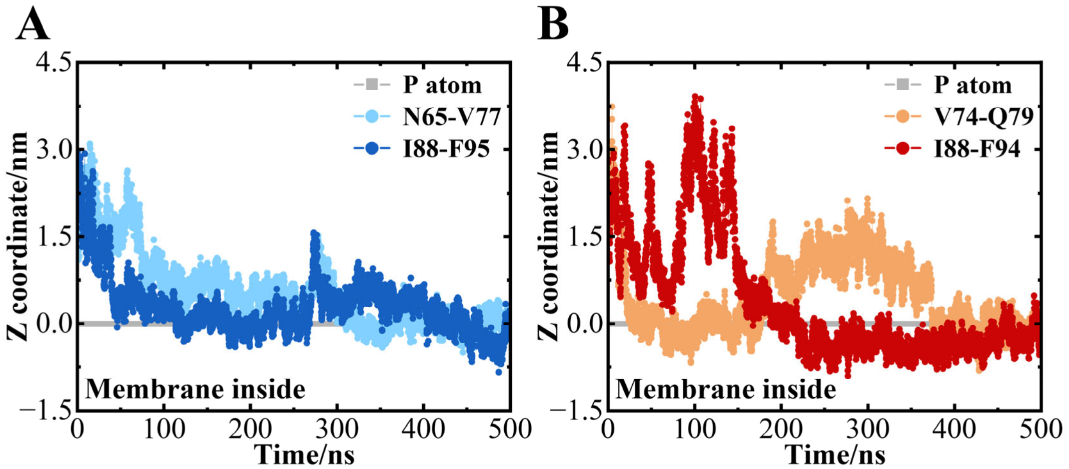

Figure S6. The time-dependent distance between the centers of mass of the two membrane interaction regions relative to the position of the phosphorus atoms (set to 0) at the membrane surface in the (**A**) absence and (**B**) presence of NaCl.

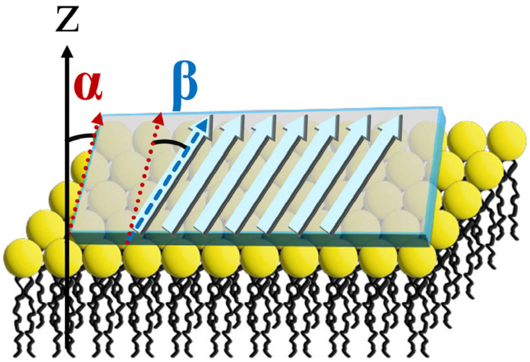

Figure S7. Schematic diagram of β-sheets bound to the membrane. Z represents the membrane normal, α is the angle between the β-sheet and Z, and β is the average orientation angle of β-strands within the sheet plane.

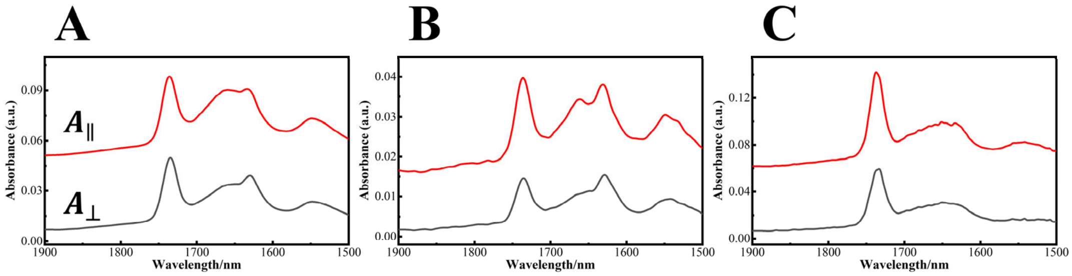

Figure S8. ATR-FTIR spectra of $\alpha S_{57\text{-}102}$ in the presence of DMPG membrane after incubation (**A**) L/P=20 in the absence of NaCl, (**B**) L/P=20 and (**C**) L/P=100 in the presence of NaCl in the 1900–1500 cm^{-1} spectral region. They measured with parallel (‖) and perpendicular (⊥) polarization of the incident beam.

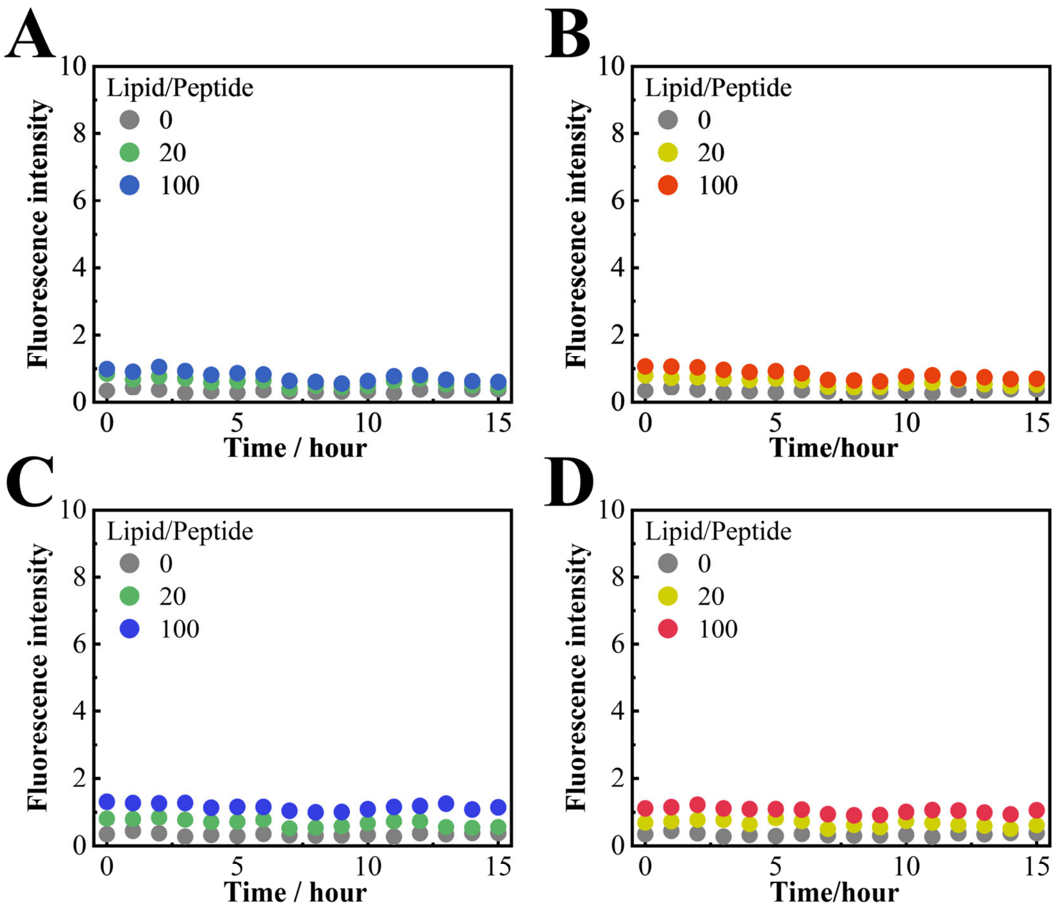

Figure S9. ThT fluorescence of background samples (lipid membrane only) under L/P ratios of 0, 20, 100 in the absence (**A**,**C**) or presence (**B**,**D**) of NaCl. The lipid membranes used were DMPG (**A**,**B**) and DMPC (**C**,**D**).
